# A Vertebral Segmentation Dataset with Fracture Grading

**DOI:** 10.1148/ryai.2020190138

**Published:** 2020-07-29

**Authors:** Maximilian T. Löffler, Anjany Sekuboyina, Alina Jacob, Anna-Lena Grau, Andreas Scharr, Malek El Husseini, Mareike Kallweit, Claus Zimmer, Thomas Baum, Jan S. Kirschke

**Affiliations:** From the Department of Diagnostic and Interventional Neuroradiology, School of Medicine, Klinikum rechts der Isar, Technical University of Munich, Ismaninger Str 22, Munich 81675, Germany (M.T.L., A. Sekuboyina, A.J., A.L.G., A. Scharr, M.E.H., M.K., C.Z., T.B., J.S.K.); and Department of Informatics, Technical University of Munich, Munich, Germany (A. Sekuboyina).

## Abstract

Published under a CC BY 4.0 license.

*Supplemental material is available for this article*.

**Summary**

This dataset provides vertebral segmentation masks for spine CT images and annotations of vertebral fractures or abnormalities per vertebral level; it is available from *https://osf.io/nqjyw/* and is intended for large-scale machine learning aimed at automated spine processing and fracture detection.

**Key Points**

■ This public CT dataset holds 160 image series of 141 patients including segmentation masks of 1725 fully visualized vertebrae; it is split into a training dataset (80 image series, 862 vertebrae), a public validation dataset (40 image series, 434 vertebrae), and a secret test dataset (40 image series, 429 vertebrae, to be released in December 2020).
■ Metadata include annotations of vertebral fractures using the semiquantitative method by Genant and of instances of foreign material per vertebral level, as well as opportunistic measurements of lumbar bone mineral density per patient.
■ This dataset was prepared for a vertebral labeling and segmentation challenge hosted at the 2019 International Conference on Medical Image Computing and Computer Assisted Intervention.

## Introduction

Automatic image analysis of the spine often requires the identification and segmentation of vertebrae before pathologies can be assessed (1–3). Several methods have been proposed to automatically assess vertebral fractures (4) or bone mineral density (BMD) (5–7). Underdiagnosis of vertebral fractures is a worldwide problem, as up to 85% of osteoporotic vertebral fractures are missed on CT scans (8). Given the abundance of CT examinations in recent years and a disproportionate increase in workload for radiologists (9), an opportunity lies in the ancillary detection of vertebral fractures on CT scans by computer-aided diagnosis. The benefits of computer-aided diagnosis in radiology have been demonstrated for other anatomic regions, like chest imaging and neuro-oncology (10,11).

Recent advances in computational performance and data processing capacity have promoted deep learning. Unlike traditional machine learning algorithms, which depend on predefined engineered features (12,13), deep learning acquires an optimal feature representation for any given task directly from the input data. In the form of convolutional neural networks (CNNs), deep learning has been successfully applied to spine segmentation tasks (1,14–16). However, deep learning methods often require a large amount of data with corresponding metadata to train models properly. Development processes become quite efficient once such data have been acquired (17). In the context of spine image analysis, such a dataset is lacking. To our knowledge, only small public CT datasets exist with vertebral segmentations of the thoracolumbar spine (Computational Spine Imaging 2014 Workshop, *n* = 20 [2,18]) and of the lumbar spine (online challenge xVertSeg, *n* = 25 [19] and a lumbar vertebra dataset, *n* = 10 [20]). Neither dataset includes cervical spine data.

We introduce a freely available CT dataset of 160 image series. Split into training and testing subsets, this dataset was used for the VerSe 2019 challenge held during the 22nd International Conference on Medical Image Computing and Computer Assisted Intervention (MICCAI) (*https://verse2019.grand-challenge.org*). Moreover, semiquantitative fracture gradings per vertebral level and opportunistic BMD measurements of the lumbar spine are provided.

## Materials and Methods

### Patients and Image Acquisition

The local institutional review board approved this retrospective evaluation of imaging data and waived written informed consent (proposal 27/19 *S*-SR). All imaging data were selected from two retrospective studies. Inclusion criteria for the first study was the availability of a lumbar dual-energy x-ray absorptiometry and a CT scan, including the lumbar region, both performed within 1 year; inclusion criteria for the second study was the availability of a nonenhanced CT scan of the entire spine. For both studies, patient selection criteria were age older than 30 years and no history of bone metastases. Imaging requirements were the availability of a 120-kVp acquisition with sagittal reformations reconstructed by filtered back projection favoring sharpness over noise (bone kernel) with a spatial resolution of at least 1 mm in the craniocaudal direction. Using these criteria, we identified 295 patients for study one (17 patients excluded due to bone metastasis) and 159 patients for study two (no patients with bone metastasis included). Of these 454 patients, we randomly selected 160 CT image series of 141 patients that satisfied our imaging requirements. All included image series have been obtained between January 2013 and November 2017. Imaging was performed in inpatients for various indications not related to bone densitometry: acute back pain or suspected spinal fracture; cancer staging, restaging, or follow-up; exclusion of acute abdominal pathology; chronic back pain; and postoperative examination. Due to scanner protocol, some patient scans of a single time point are subdivided into two or three image series (eg, cervical, thoracic, and lumbar stack), which represent separate data entities. There was an overlap of 15 patients with a previous study investigating the association of lumbar BMD with incident vertebral fractures (21).

### CT Imaging

CT scans were performed with five multidetector CT scanners (Philips Brilliance 64, iCT 256, and IQon, Philips Medical Care; Siemens Somatom Definition AS and AS+; Siemens Healthineers); some scans were performed after administration of either both oral (Barilux Scan; Sanochemia Diagnostics) and intravenous (Iomeron 400; Bracco) contrast medium or only intravenous contrast material. Image data were acquired with all scanners in helical mode with a peak tube voltage of 120 kVp, a slice thickness of 0.9–1 mm, and adaptive tube load. Postcontrast scans were acquired either in the arterial or portal venous phase, triggered by a threshold of CT attenuation surpassed in a region of interest placed in the aorta or after a delay of 70 seconds, respectively.

### Vertebral Segmentation

**Figure 1:**
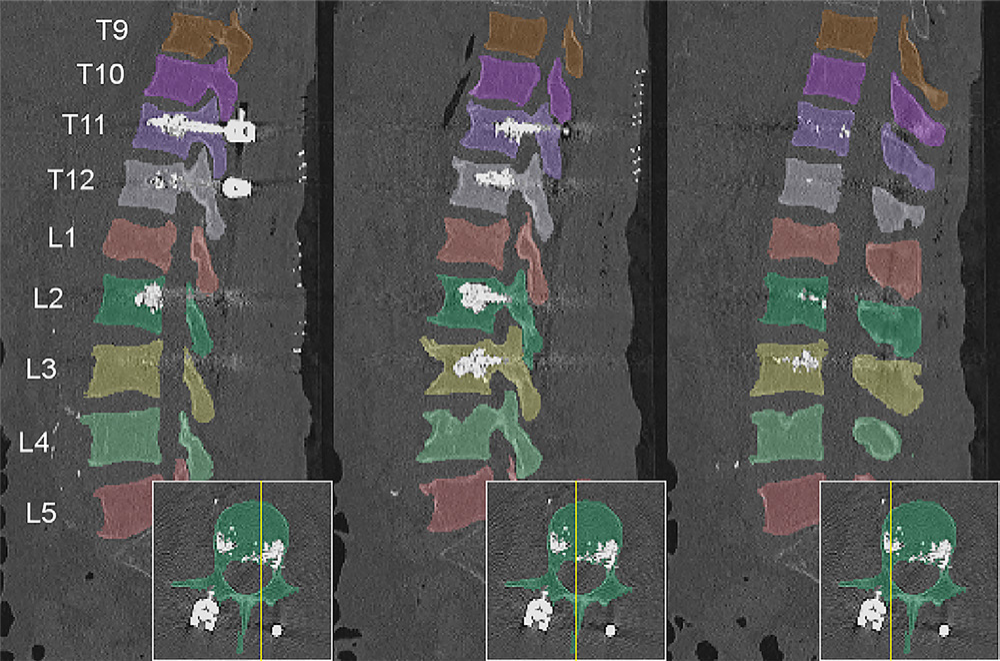
Sagittal reformations of an example CT scan in the dataset with segmentation mask visualized as colored overlays. This patient had internal fixation of vertebral levels T11 through L3 augmented with intravertebral polymethyl methacrylate.

Segmentation masks of vertebrae were generated in a three-step approach. First, CT data were anonymized by conversion to Neuroimaging Informatics Technology Initiative (NIfTI) format (*https://nifti.nimh.nih.gov/nifti-1*) and reduced in resolution to limit computational demands for deep learning algorithms. This resulted either in image series of 1-mm isotropic resolution or in sagittal 2-mm to 3-mm series of 1-mm in-plane resolution. Second, we implemented a framework to predict accurate voxel-level segmentations of the vertebrae (16). This framework used a fully CNN to detect the spine resulting in a low-resolution heatmap, a Btrfly Net to label vertebrae on sagittal and coronal maximum intensity projections (22,23), and an improved U-Net to segment vertebral patches centered around vertebral labels at original resolution (24). Vertebral patches are fused to one segmentation mask labeled by vertebral level. The U-Net was initially trained with public datasets (Computational Spine Imaging and xVertSeg) and was continuously retrained with finalized segmentation masks of this dataset. Third, segmentation masks were manually refined by one of four specifically trained medical students (A.J., A.L.G., A. Scharr, M.K.) and thereafter by one of two neuroradiologists (M.T.L. and J.S.K.) using the open-source software ITK-SNAP (25). Any material not physiologically related to bone mineral and extracellular matrix (ie, screw-rod systems, intervertebral cages, and intravertebral polymethyl methacrylate for vertebroplasty or screw augmentation) was excluded (Fig 1).

### Assessment of Vertebral Fractures and BMD

All CT scans were evaluated for prevalent fractures and foreign material at each vertebral level. Only thoracolumbar vertebrae were evaluated, as fractures are rare and usually of nonosteoporotic origin at the cervical spine. Foreign material included polymethyl methacrylate–augmentation and implants for internal fixation and spinal fusion. Image assessment was performed in consensus by two radiologists (M.T.L. and J.S.K.), with 5 years and 17 years of experience, respectively. Prevalent vertebral fractures were classified using the semiquantitative method by Genant et al (26). Briefly, vertebral fractures were graded as mild for a height loss ≥ 20% and < 25%, as moderate for a height loss of ≥ 25% and < 40%, and as severe for a height loss ≥ 40%. The type of fracture was categorized into wedge (anterior height loss most prominent), biconcave (central height loss most prominent with almost equal anterior and posterior height loss), or crush (posterior height loss most prominent or uniform height loss including the posterior vertebral wall) fracture. Deformities and developmental abnormalities, like in Scheuermann disease, were not graded as fractures.

Opportunistic screening of lumbar BMD was performed in all patients using asynchronous calibration (21). In case of unenhanced scans, BMD quantification with asynchronously calibrated CT can be considered equal to classic quantitative CT (27).

### Statistical Analysis

Means of continuous variables (age and BMD) were compared with independent two-sample *t* test. Proportions of categorical variables (sex, intravenous contrast agent, CT scanner) were compared with Pearson χ^2^ test. Level of significance was defined at *P* < .05. Statistics were calculated with IBM SPSS Statistics 24 (IBM, Armonk, NY).

### Resulting Dataset

To generate this dataset, a total of 141 patients were included, with 160 CT image series and 1725 vertebrae encompassing 220 cervical, 884 thoracic, and 621 lumbar vertebrae (Table). This represents a more than fourfold increase in available annotated data—in particular for pathologic and cervical vertebrae—compared with previously available datasets with vertebral segmentations (2,20–22). The patients had a mean age of 66.1 years ± 15 (standard deviation) including 49 men (59.8 years ± 16.6) and 92 women (69.4 years ± 12.9). Most patients presented with a low BMD (77.8 mg/cm^3^ ± 53.6), while women had a significantly lower BMD compared with men (63.4 mg/cm^3^ ± 44.1 vs 104.9 mg/cm^3^ ± 59.5, *P* < .001). Ninety-one patients had at least one osteoporotic vertebral fracture; patients with fractures were significantly older and had lower BMD compared with those without fractures (69.5 years ± 13.1 vs 56.6 years ± 17.2 and 58.7 mg/cm^3^ ± 40.8 vs 115.3 mg/cm^3^ ± 59.5, each *P* < .001). Patient characteristics (sex, age, BMD, contrast media applied, scanner used) were not significantly different between training and both test datasets (each *P* > .05; Table). Of note, CT image series of one patient are contained within one dataset. The number of included and fractured vertebrae per level is depicted in two diagrams (Figs E1, E2 [supplement]). Wedge type and grade 1 fractures predominated (Fig E3 [supplement]). Patients in their seventies and with osteoporotic BMD (lower than 80 mg/cm^3^) represented the largest groups (Fig E4 [supplement]).

**Table:**
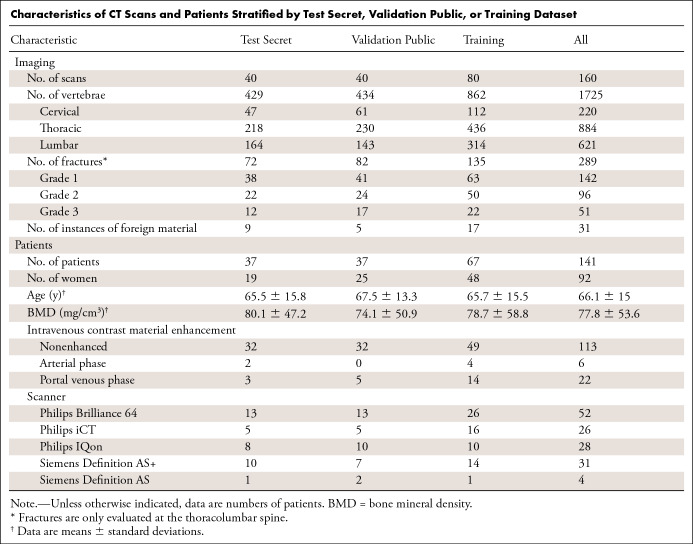
Characteristics of CT Scans and Patients Stratified by Test Secret, Validation Public, or Training Dataset

**Figure 2:**
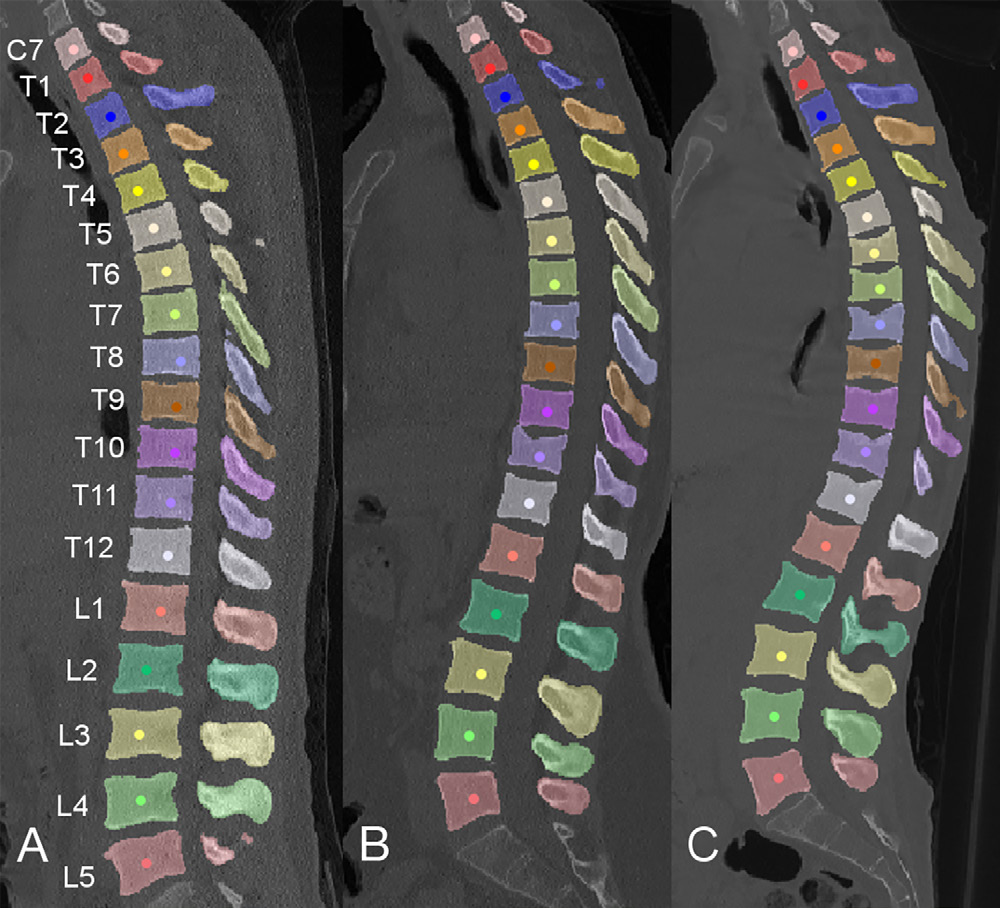
Example segmentations that can be found in the dataset with masks visualized as colored overlays and approximate centroid labels as colored points. Images show, *A,* a case without fracture, *B,* a patient with an osteoporotic fracture of T11, and, *C,* a patient with multiple osteoporotic fractures.

Published under the creative commons license CC BY-SA 4.0, the data are hosted at the open science framework (*https://osf.io/nqjyw**/*). For the purpose of the labeling and segmentation challenge held at MICCAI 2019, the CT data (NIfTI format) are separated into training (80 image series, 862 vertebrae), public validation (40 image series, 434 vertebrae), and secret test data (40 image series, 429 vertebrae, to be released in December 2020). For training data, accompanying segmentation masks (NIfTI format) and labels of all segmented vertebrae (JavaScript Object Notation [JSON] format) are provided (Fig 2). Additionally, we provide the fracture classification for each vertebra in a spreadsheet (Appendix E1 [supplement]).

### Limitations and Future Work

This public dataset had a few limitations. We only included patients older than 30 years; therefore, algorithms trained with this data could render less reliable results for younger individuals. There are many normal variants and vertebral abnormalities that are not covered by this dataset (eg, we excluded bone metastasis and primary bone tumors). Several postoperative changes including polymethyl methacrylate and screw-rod systems are present in both training and test sets, but a rigorous evaluation and inclusion of all postoperative changes possible (including vertebral replacements) is still missing. Additionally, we focused on edge-enhancing reconstructions, as these are usually the reconstructions used for interpretation of bony structures at CT; however, it would also be interesting to include soft-tissue kernels and iterative reconstruction algorithms. Also, due to the retrospective design of this data collection, isotropic resolution was not available in all scans. We also had to limit the spatial resolution to 1 mm in each direction, as a manual correction of, for example, 0.5-mm isotropic reconstructions, would increase the workload of the manual corrections eightfold compared with our approach. An isotropic resolution of 1 mm was thought to be the best compromise between still depicting clinically relevant structures and manageable workload in a large number of patients. However, for the cervical spine of small patients, higher spatial resolution may be wanted.

Another point of discussion is the correctness of the presented segmentation masks. Notwithstanding the bias introduced by the automatic approach, the final go-ahead was given by a single rater. Adding multiple raters will result in variability in the masks. Therefore, a multirater fusion of annotations might be also of interest. Third, the inclusion of degenerative changes makes it impossible, in some cases, to draw the correct border between two fused vertebrae or some low-density degenerative calcification and the adjacent soft tissue, for example. On low-quality scans with a lot of background noise, this differentiation can become difficult.

Of note, vertebral segmentation and morphometry is also of interest using MRI data (28). Future work could address training and validation of automated segmentation algorithms in MRI.

Results from the VerSe 2019 challenge at the MICCAI conference showed that machine learning algorithms proposed by the participants can achieve accurate and reliable automated spine segmentation. The winning algorithm scored Dice coefficients around 0.9 (16,29). Moreover, with this dataset algorithms for automated fracture detection can be trained and validated. Future work will be needed to demonstrate if patients can benefit from computer-aided diagnosis, which would support radiologists in the detection of spine pathology.

## APPENDIX

Appendix E1 (PDF)

## SUPPLEMENTAL FIGURES

Figure E1:

Figure E2:

Figure E3:

Figure E4:
