# Supplementary figures and images for: A Vertebral Segmentation Dataset with Fracture Grading
Source: Radiol Artif Intell. 2020 Jul 29;2(4):e190138. doi: 10.1148/ryai.2020190138 (PMC8082364; doi:10.1148/ryai.2020190138)

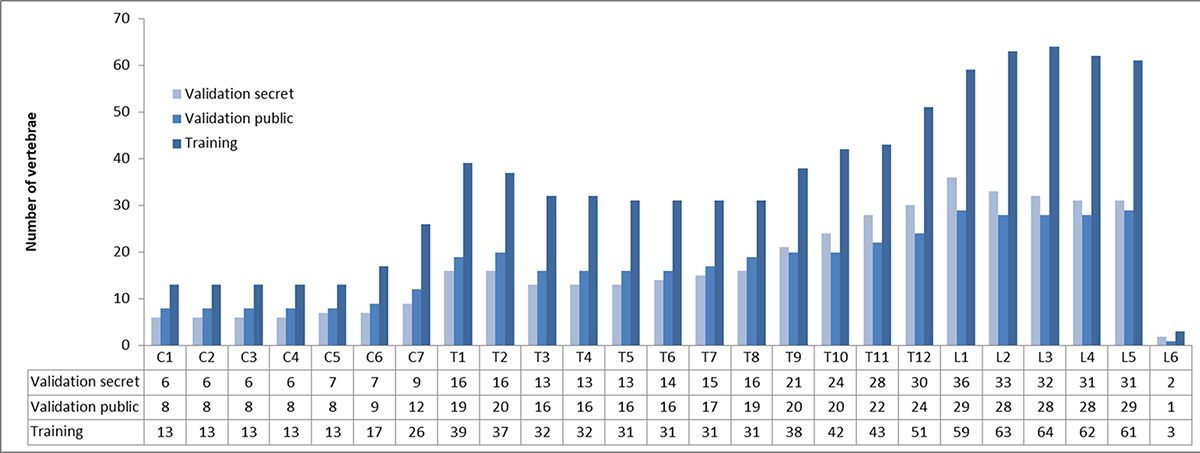

Supplement: Figure E1: [file ryai190138suppf1.jpg]

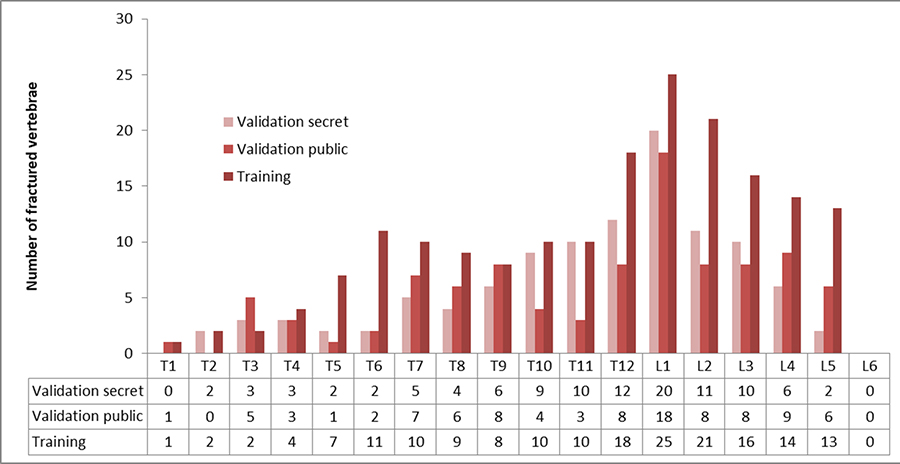

Supplement: Figure E2: [file ryai190138suppf2.jpg]

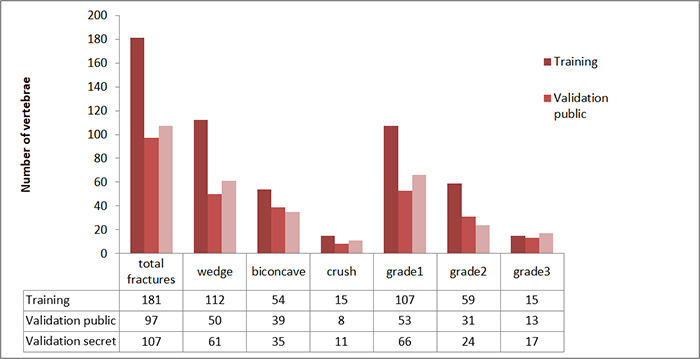

Supplement: Figure E3: [file ryai190138suppf3.jpg]

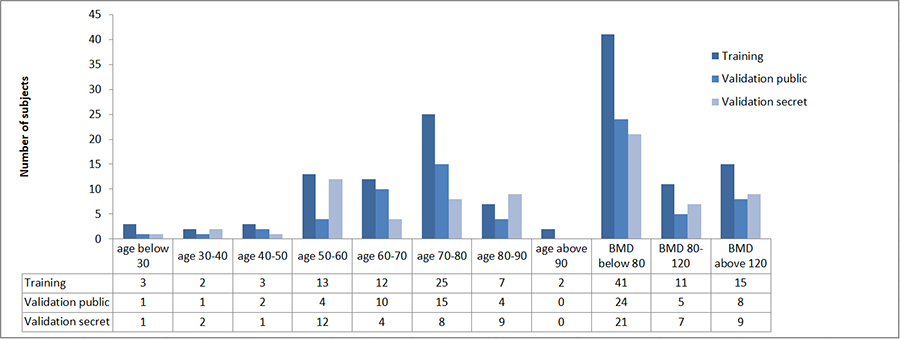

Supplement: Figure E4: [file ryai190138suppf4.jpg]
